# Biomarkers for Autism Spectrum Disorders (ASD): A Meta-analysis

**DOI:** 10.5041/RMMJ.10375

**Published:** 2019-10-29

**Authors:** Ashley Ansel, Yehudit Posen, Ronald Ellis, Lisa Deutsch, Philip D. Zisman, Benjamin Gesundheit

**Affiliations:** 1 Cell-El Therapeutics Ltd, Jerusalem, Israel; 2 PSW Ltd, Rehovot, Israel; 3 Biotech & Biopharma Consulting, Jerusalem, Israel; 4 Biostats Statistical Consulting Ltd, Modiin, Israel

**Keywords:** Autism spectrum disorder, biomarkers, gene expression, magnetic resonance imaging, meta-analysis, proteomics

## Abstract

**Objective:**

To compare the reported accuracy and sensitivity of the various modalities used to diagnose autism spectrum disorders (ASD) in efforts to help focus further biomarker research on the most promising methods for early diagnosis.

**Methods:**

The Medline scientific literature database was searched to identify publications assessing potential clinical ASD biomarkers. Reports were categorized by the modality used to assess the putative markers, including protein, genetic, metabolic, or objective imaging methods. The reported sensitivity, specificity, area under the curve, and overall agreement were summarized and analyzed to determine weighted averages for each diagnostic modality. Heterogeneity was measured using the *I^2^* test.

**Results:**

Of the 71 papers included in this analysis, each belonging to one of five modalities, protein-based followed by metabolite-based markers provided the highest diagnostic accuracy, each with a pooled overall agreement of 83.3% and respective weighted area under the curve (AUC) of 89.5% and 88.3%. Sensitivity provided by protein markers was highest (85.5%), while metabolic (85.9%) and protein markers (84.7%) had the highest specificity. Other modalities showed degrees of sensitivity, specificity, and overall agreements in the range of 73%–80%.

**Conclusions:**

Each modality provided for diagnostic accuracy and specificity similar or slightly higher than those reported for the gold-standard Autism Diagnostic Observation Schedule (ADOS) instrument. Further studies are required to identify the most predictive markers within each modality and to evaluate biological pathways or clustering with possible etiological relevance. Analyses will also be necessary to determine the potential of these novel biomarkers in diagnosing pediatric patients, thereby enabling early intervention.

## INTRODUCTION

Autism spectrum disorders (ASD) were first characterized clinically in 1943 by Kanner^1^ and further in 1979 by Wing and Gould^2^ as a spectrum of impaired social interactions, restricted communications skills, and unusual repetitive behaviors. The American Psychological Association’s *Diagnostic and Statistical Manual of Mental Disorders, Fifth Edition* (DSM-5) recently consolidated the various subtypes of pervasive developmental disorders (PDD) into one category called ASD and shifted the evaluation from three domains (social deficits, communication deficits, and restricted repetitive behaviors [RRB]) to two (social-communication impairments and RRB).^3^

Several behavior assessment-based diagnostic tests have been used for ASD, including the Autism Diagnostic Observation Schedule (ADOS).^4^ A meta-analysis involving ADOS evaluations of >4,000 children reported an overall diagnostic accuracy of 52%, with sensitivity scores of 67%–97% (pooled data: 91%) and specificity scores of 56%–94% (pooled data: 73%).^5^ The Autism Diagnostic Interview–Revised (ADI-R) is a structured interview of the parent, differing from the direct observation of the child performed with the ADOS evaluation.^6^ In addition, clinicians use the Childhood Autism Rating Scale (CARS) to rate the child’s behavior on 15 subscales;^7^ parental reports can also be considered.

Since these behavioral tests are subjective and time-consuming, require professional staff to be administered, and can only be used from age 3 years once the child is old enough to communicate, researchers have sought other ways of diagnosing ASD.^8^ Biomarkers are expected to be more objective, should enable earlier diagnosis, and may provide clues to the underlying etiology of ASD. In addition, providing positive diagnosis in younger toddlers may enable earlier initiation of therapy with consequently higher probability of successful treatment given decreasing brain plasticity with age in the developing child. The primary modalities harnessed to identify novel ASD biomarkers have been molecular, proteomic, metabolomic, neurochemical, radiologic, and electrophysiologic, with transcriptomic analyses also having been performed.^9^

This study aimed to compare the reported accuracy and sensitivity of the various modalities used to diagnose ASD. This should help focus further biomarker research on the most promising methods for early diagnosis.

## METHODS

This analysis aimed to adhere to the Preferred Reporting Items for Systematic Reviews and Meta-Analyses (PRISMA) guidelines, to ensure comprehensive and transparent reporting.

### Search Strategy

Independent queries using medical subject headings (MeSH) and keywords were performed to identify all primary research articles from the PubMed database that evaluated sensitivity, specificity, and accuracy of biomarkers for diagnosing ASD within six predefined modalities (protein, metabolic, genetic, electroencephalography [EEG], magnetic resonance imaging [MRI], positron emission tomography [PET]) (Supplement). Each search term included autism/ASD, a diagnostic technique, and a sensitivity/specificity classifier. To distinguish between protein and metabolic papers, all papers dealing with hormones, urine, mass spectrophotometry, metabolites, or peptides were assigned to the metabolic modality, while all papers dealing with cytokines, chemokines, or other proteins circulating in the blood were assigned to the protein modality.

### Data Sources and Data Extraction

The search terms were entered into Ovid MEDLINE (1946 to January 2017) without limits, and 866 articles were returned. Reviews and reference lists were cross-checked for studies that the search terms might have missed.

#### Screening

Two independent reviewers (AA and JR) examined study titles. From review of the abstracts, potentially eligible full-text articles were retrieved with relevant appendices and supplementary information.

#### Eligibility

Full-text articles were reviewed against eligibility criteria. Inclusion criteria were: (a) articles after 1994, written in English; (b) inclusion of a typically developing (TD) control group (unless it was a review paper); (c) inclusion of a study group with children diagnosed with ASD by a behavioral diagnostic test or by DSM criteria (unless it was a review paper); and (d) assessment for ASD biomarkers using one of the six predefined modalities. Exclusion criteria were: (a) studies that compared ASD with other comorbidities; (b) studies that tested only risk factors of ASD; (c) studies that had a therapeutic component; (d) non-clinical studies; and (e) studies without statistical parameters of interest (sensitivity, specificity, accuracy, and/or area under the curve [AUC]). *All* publications meeting all inclusion criteria and none of the exclusion criteria were included in the analysis.

#### Data Extraction

For each eligible article, the following data were extracted and validated independently by two researchers (AA and JR): first author’s surname, year of publication, diagnostic modality, number of ASD subjects, number of controls, age-matched (yes or no), sex-matched (yes or no), accuracy (% correctly identified), AUC, sensitivity, and specificity. The standard error of the AUC was calculated based on the AUC point estimate and sample size by the method of Hanley and McNeil.^10^ Several papers included the evaluation of multiple markers assessed on the same group of subjects; these were included in the meta-analysis individually and analyzed independently of each other.

#### Data Analysis

A separate statistical analysis of each of the parameters was performed for each of the six predefined modalities. Meta-analyses of sensitivity, specificity, AUC, and accuracy were performed using MedCalc Statistical Software version 17.9.7 (MedCalc Software bvba, Ostend, Belgium). The weighted summary sensitivity, specificity, accuracy (arc-sine square root transformation), and AUC with 95% confidence intervals (CIs) were calculated using a random-effects model.^11^ Inter-study heterogeneity was assessed using the *I^2^* statistic with 95% CI, which describes the percent variability in point estimate due to heterogeneity rather than sampling error. Presented are the weighted summaries of each tested parameter, alongside the weighted summary of the ADOS test, as reported in a published meta-analysis.^5^ The contribution of each individual publication to the weighted summary of each measured parameter is presented by modality.

## RESULTS

**Figure 1:**
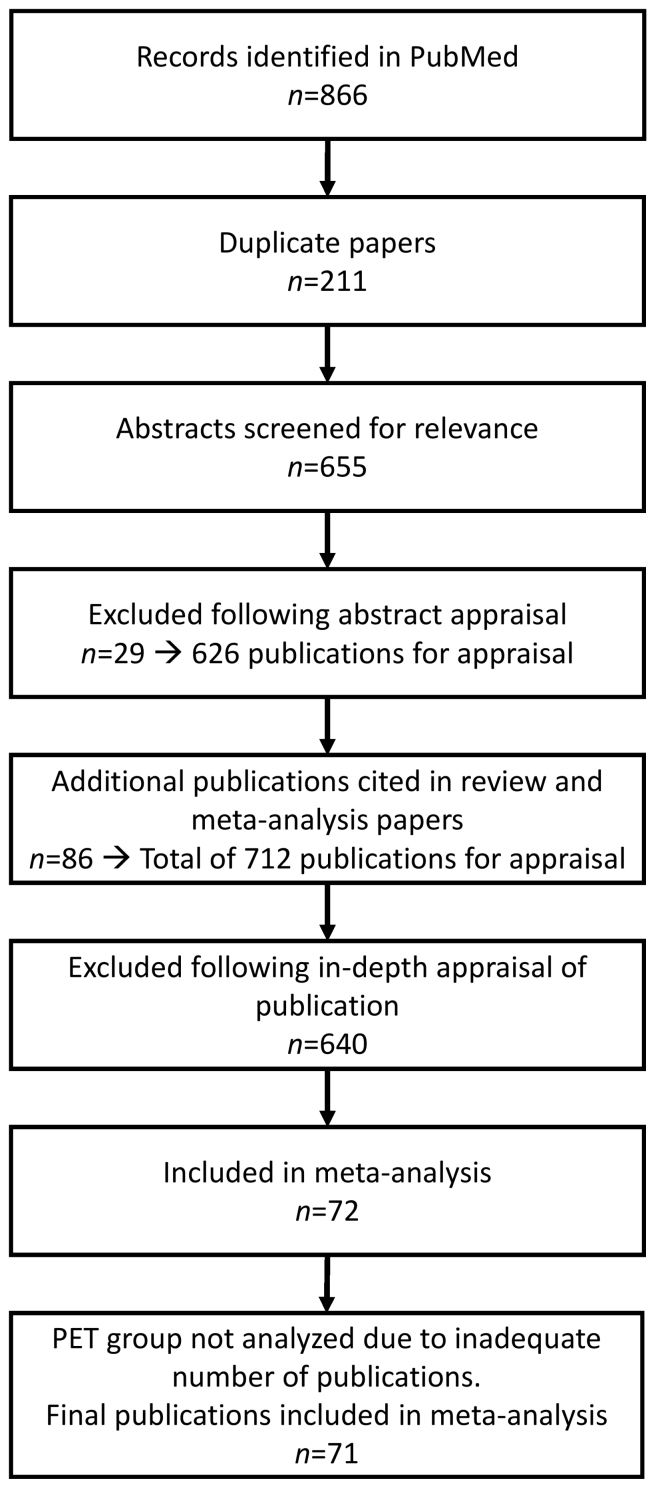
PRISMA Flow Diagram of the Phases of the Literature Search.

The literature search identified 866 papers (Figure 1), of which 211 were duplicates. The abstracts of the remaining 655 papers were screened for relevance; 29 were excluded. Thus, 626 full-text papers were assessed for eligibility based on the above criteria. Review of these papers identified 86 additional papers which were also further appraised. Of these 712 publications in total, 640 failed to meet the inclusion criteria, primarily due to lack of an ASD group or insufficient statistical data. The 72 papers that met the criteria were subdivided by the diagnostic modality reported in the study. Four papers included data belonging to two diagnostic modalities.^12^–^15^ Since only one PET study met the eligibility criteria, this modality was excluded from the analysis. Cohort sizes varied from 6 to 554 subjects.

Genetic studies (*n*=14)^13^,^14^,^16^–^27^ applied polymerase chain reaction (PCR) genotyping, mRNA/miRNA microarray, or spectrophotometric tools. Magnetic resonance imaging (MRI) studies (*n*=22),^13^,^14^,^21^,^28^–^46^ which included functional MRI (fMRI), resting state fMRI (rs-fMRI), structural MRI (sMRI), and standard MRI studies, sought out ASD diagnostic markers by analyzing fast spin echo (FSE) T2-weighted, fluid-attenuated inversion recovery (FLAIR), diffusion-weighted imaging (DWI), spin echo (SE) T1-weighted sequences, and single voxel 1H MR spectrum. Volumetric measurements for different areas of the brain were assessed. Mass univariate methods such as voxel-based morphometry (VBM) and whole-brain classification approach employing a support vector machine (SVM) were used. In the 21 metabolic studies,^12^,^15^,^47^–^65^ high-performance liquid chromatography (HPLC), liquid chromatography–high resolution mass spectrometry (LC-HRMS), gas chromatography, nuclear magnetic resonance (NMR), as well as capillary electrophoresis with ultraviolet/ visible spectroscopy (UV-Vis) were used to identify markers. The 12 studies assessing the diagnostic potential of various protein markers^12^,^15^,^66^–^75^ covered >60 proteins or protein combinations using various microarray kits/chips or enzyme-linked immunosorbent assay (ELISA). Six studies focused on EEG analyses for ASD diagnosis.^76^–^81^

**Figure 2:**
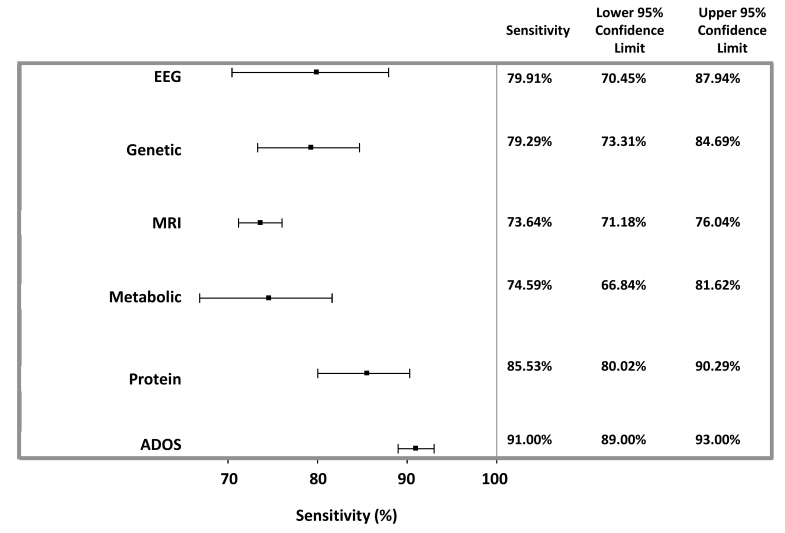
Weighted Sensitivity of Appraised Studies. The weighted sensitivity with 95% CIs was calculated using a random-effects model. Also shown is the weighted sensitivity of the ADOS test, as determined in a meta-analysis of seven cross-sectional studies assessing >4,000 children.^5^

**Figure 3:**
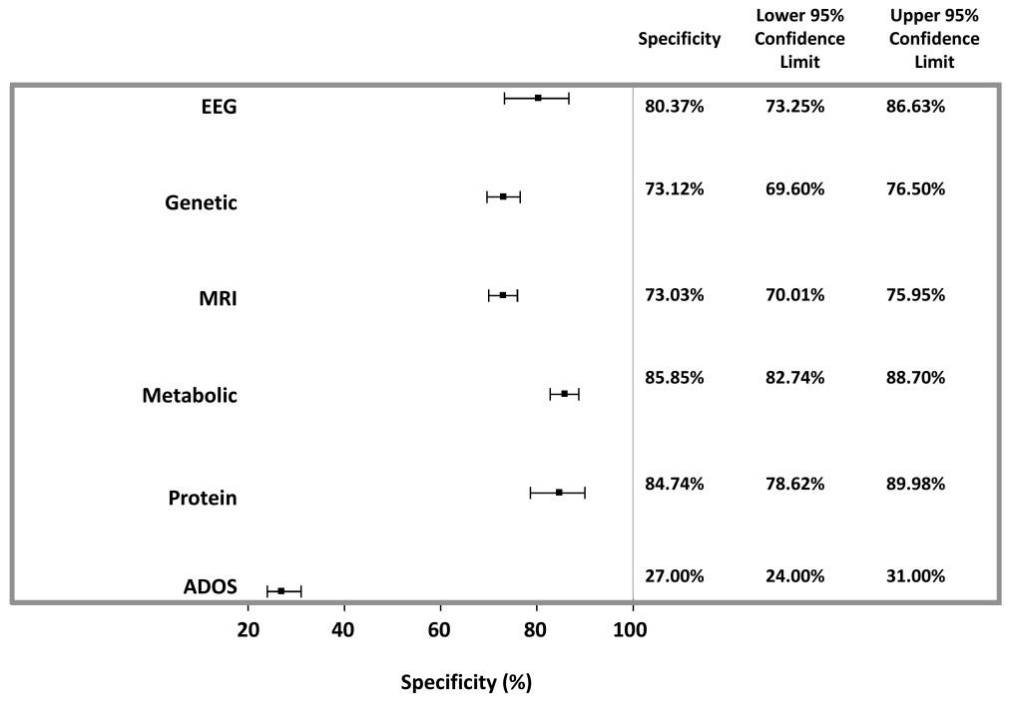
Weighted Specificity of Appraised Studies. The weighted specificity with 95% CIs was calculated using a random-effects model. Also shown is the weighted sensitivity of the ADOS test, as determined in a meta-analysis of seven cross-sectional studies assessing >4,000 children.^5^

**Figure 4:**
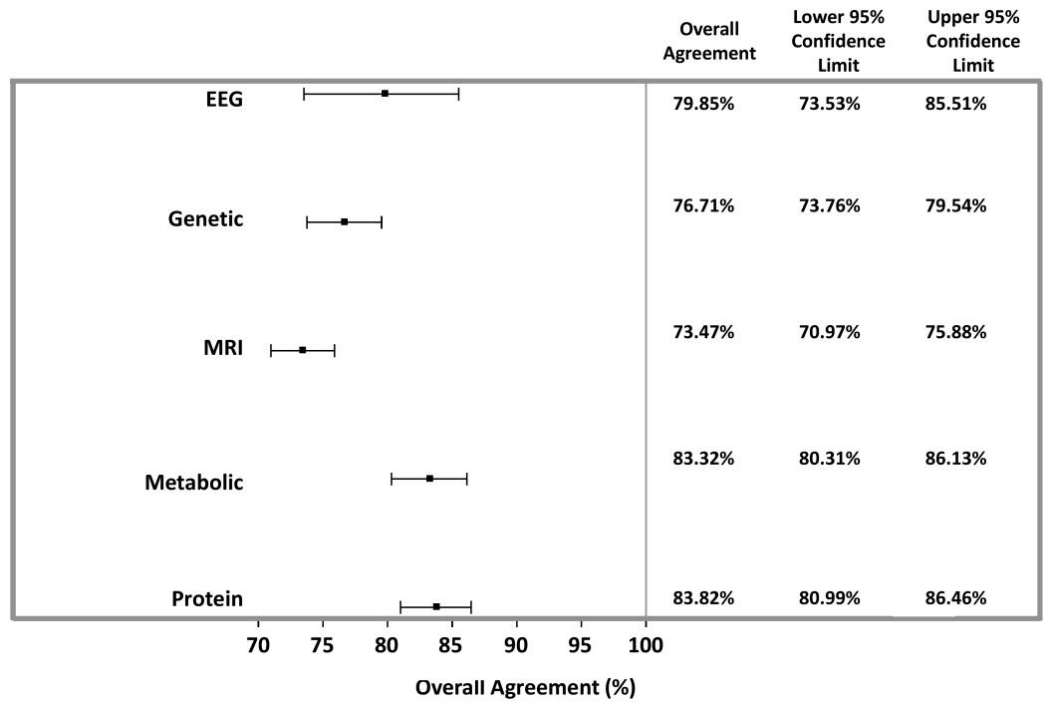
Weighted Overall Agreement of Appraised Studies. The weighted overall agreement with 95% CIs was calculated using a random-effects model.

**Figure 5:**
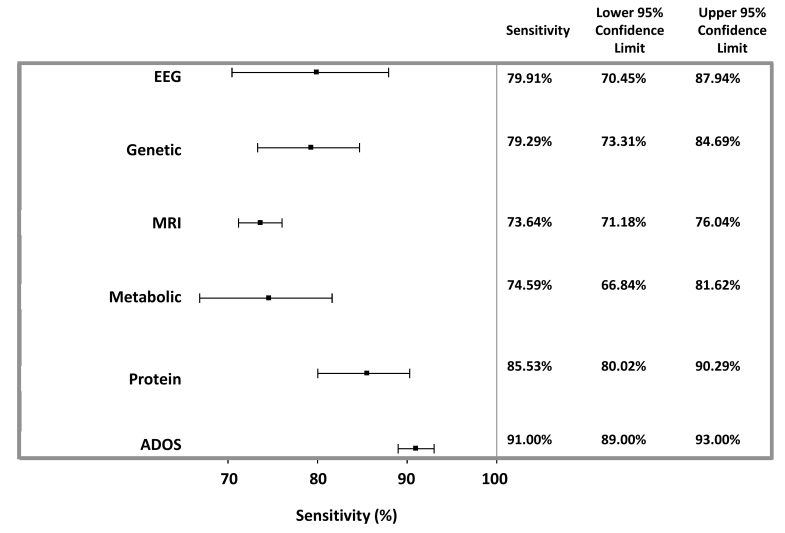
Weighted AUC of Appraised Studies. The weighted AUC with 95% CIs were calculated using a random-effects model.

Overall, protein-based followed by metabolite-based studies provided the highest diagnostic accuracy, each with an overall agreement of 83.3% and AUC of sensitivity (true-positive rates) versus specificity (false-positive rates) of 89.5% and 88.3%, respectively (Table 1, Figures 2–5). Sensitivity provided by protein markers (85.5%) and metabolite markers (84.7%) was highest. The other modalities showed similar degrees of sensitivity, specificity, and overall agreements, which all fell within the range of 73%–80%.

**Table 1:** Weighted ASD Diagnostic Power for Each Evaluated Modality.

Modality	Sensitivity (%)	Specificity (%)	Overall Agreement (%)	Area under Curve (AUC) (%)
**Genetic**				
Pool	79.3	73.1	76.7	79.5
Range	24.3–100	41.8–88.2	62.7–90.4	64.8–92.0
95% CI	73.3–84.7	69.6–76.5	73.8–79.5	77.0–82.0
*I^2^*	87.5	56.9	70.8	52.5

**MRI**				
Pool	73.6	73.0	73.5	79
Range	43.7–94.8	45.4–100	57.9–95.7	58.0–99.5
95% CI	71.8–76.0	70.0–76.0	71.0–75.9	75.0–83.0
*I^2^*	78.1	84.4	89.1	96.4

**Metabolic**				
Pool	74.6	85.9	83.3	88.3
Range	0–100	15.0–100	48.7–100	59.2–99.9
95% CI	66.8–81.6	82.7–88.7	80.3–86.1	86.0–91.0
*I^2^*	97.7	87.1	ND	92.2

**Protein**				
Pool	85.5	84.7	83.8	89.5
Range	50–100	38.9–100	62.8–100	57.0–99.9
95% CI	80.0–90.3	78.6–90	81.0–86.5	86.0–93.0
*I^2^*	87.3	89.2	73	90.1

**EEG**				
Pool	79.9	80.4	79.9	ND
Range	57.6–90.9	64.5–100	70.5–87.4	ND
95% CI	70.5–87.9	73.3–86.6	73.5–85.5	ND
*I^2^*	83.4	79.5	85.2	ND

ND, not done.

## DISCUSSION

As the first reported meta-analysis of ASD biomarkers, the current study included 71 papers with cohorts of up to 554 ASD subjects.^77^ This study suggests that all five major bio-diagnostic modalities provide similar diagnostic objective accuracy for ASD compared to the subjective ADOS. The protein- and metabolite-based tests were found to provide for the highest diagnostic accuracy; combining modalities might further improve diagnostic accuracy.

The sensitivities of the various studied modalities were 73.6%–85.5%, with protein markers showing the highest degree of sensitivity. The specificities ranged from 73.0% (MRI) to 85.9% (metabolic). Accuracy, assessed by overall agreement and AUC, was 73.4%–83.2% and 79.0%–89.5%, respectively, and highest for protein-based biomarkers. Taken together, all analyzed modalities provided for higher diagnostic accuracy and specificity compared to the gold-standard ADOS test.^5^ While pooled ADOSdiagnostic sensitivity was higher than for biomarker modalities, protein-based diagnoses provided for sensitivity within the same range. This meta-analysis supports efforts to search for/use new objective modalities beyond psychological tests for the diagnosis of ASD. Moreover, quantitative objective biomarkers identified at ages when psychological tests cannot yet be employed should enable earlier-stage intervention, which is projected to be more efficient due to greater brain plasticity. These diagnostic efforts may enable the subdivision of ASD into subgroups and provide useful therapeutic targets, which have significant long-term therapeutic implications.

Further studies will be necessary to determine which modalities serve better as screening versus confirmatory testing. Subgroups of ASD might be defined by these tests, suggesting different therapeutic modalities as diagnostic targets for different such subgroups. Attempts to find patterns linking the most accurate biomarkers in each modality may identify common pathways and draw the ASD community closer to developing therapeutics, where these diagnostic markers will serve with psychological tests as objective theragnostic monitoring tools.

There were several limitations in this meta-analysis. There was a high level of heterogeneity, as expected given both the clinical heterogeneity between the included papers, with variations in the diagnosis and definition of ASD, and the methodologies across studies. In addition, in all assessed publications, evaluation of the diagnostic value of the biomarker of interest used typically developing controls as a comparator group, which may falsely elevate the diagnostic capacity of the test as compared to its performance in marginal cases with behaviors consistent with ASD. Comparing across modalities was not uniformly well-controlled. In addition, certain modalities were documented in a very limited number of papers. Furthermore, many papers were excluded due to insufficient statistical data, reinforcing the importance of proper study design and execution in future analyses of ASD biomarkers.

In conclusion, this study suggests that five major bio-diagnostic modalities provide a higher level of accuracy for objective diagnosis of ASD compared to ADOS, the gold-standard test. Thus, it is justified to include objective biological tests in the diagnosis of ASD to develop and monitor future biological therapies. Further studies looking at each modality in higher resolution to fine-tune the findings are still necessary. Objective biomarkers together with current psychological evaluations might enable improved diagnosis and monitoring.

## Supplementary Data



## Abbreviations

ADI-R: Autism Diagnostic Interview–Revised
ADOS: Autism Diagnostic Observation Schedule
ASD: autism spectrum disorders
AUC: area under the curve
CARS: Childhood Autism Rating Scale
DSM: Diagnostic and Statistical Manual of Mental Disorders
EEG: electroencephalography
ELISA: enzyme-linked immunosorbent assay
fMRI: functional magnetic resonance imaging
HPLC: high-performance liquid chromatography
LC-HRMS: liquid chromatography–high resolution mass spectrometry
MRI: magnetic resonance imaging
NMR: nuclear magnetic resonance
PDD: pervasive developmental disorders
PCR: polymerase chain reaction
PET: positron emission tomography
PRISMA: Preferred Reporting Items for Systematic Reviews and Meta-Analyses
RRB: restricted repetitive behaviors
rs-fMRI: resting state functional magnetic resonance imaging
sMRI: structural magnetic resonance imaging
SVM: support vector machine
TD: typically developing
UV-Vis: ultraviolet/visible spectroscopy
VBM: voxel-based morphometry.
