# Supplementary material for: Biomarkers for Autism Spectrum Disorders (ASD): A Meta-analysis
Source: Rambam Maimonides Med J. 2019 Oct 29;10(4):e0021. doi: 10.5041/RMMJ.10375 (PMC6824829; doi:10.5041/RMMJ.10375)
Supplement: Supplementary file 1 [file rmmj-10-4-e0021-supp.pdf]

SUPPLEMENTARY MATERIAL

This appendix has been provided by the authors to give readers additional background reading and

Supplement to Biomarkers for Autism Spectrum Disorders (ASD): A Meta-analysis

Ansel A, Posen Y, Ellis R, Deutsch L, Zisman PD, Gesundheit B. Biomarkers for Autism Spectrum Disorders (ASD): A Meta-analysis. Rambam Maimonides Med J 2019;10 (4):e0021. doi:10.5041/RMMJ.10375Reference

SEARCH TERMS

1. Autism spectrum disorder OR "autism" AND "MRI" AND “sensitivity”
2. Autism spectrum disorder OR "autism" AND "MRI" AND “specificity”
3. Autism spectrum disorder OR "autism" AND "MRI" AND (“classifier” OR “classification”)
4. Autism spectrum disorder OR "autism" AND "PET" AND “sensitivity”
5. Autism spectrum disorder OR "autism" AND "PET" AND “specificity”
6. Autism spectrum disorder OR "autism" AND "PET" AND (“classifier” OR “classification”)
7. Autism spectrum disorder OR "autism" AND "cytokines" AND “sensitivity”
8. Autism spectrum disorder OR "autism" AND "cytokines" AND “specificity”
9. Autism spectrum disorder OR "autism" AND "cytokines" AND (“classifier” OR “classification”)
10. Autism spectrum disorder OR "autism" AND "chemokines" AND “sensitivity”
11. Autism spectrum disorder OR "autism" AND "chemokines" AND “specificity”
12. Autism spectrum disorder OR "autism" AND "chemokines" AND (“classifier” OR “classification”)
13. Autism spectrum disorder OR "autism" AND "protein" AND “sensitivity”
14. Autism spectrum disorder OR "autism" AND "protein" AND “specificity”
15. Autism spectrum disorder OR "autism" AND "protein" AND (“classifier” OR “classification”)
16. Autism spectrum disorder OR "autism" AND "SNP" AND “sensitivity”
17. Autism spectrum disorder OR "autism" AND "SNP" AND “specificity”
18. Autism spectrum disorder OR "autism" AND "SNP" AND (“classifier” OR “classification”)

19. Autism spectrum disorder OR "autism" AND "polymorphism" AND “sensitivity”
20. Autism spectrum disorder OR "autism" AND "polymorphism" AND “specificity”
21. Autism spectrum disorder OR "autism" AND "polymorphism" AND (“classifier” OR “classification”
22. Autism spectrum disorder OR "autism" AND "MRNA" AND “sensitivity”
23. Autism spectrum disorder OR "autism" AND "MRNA" AND “specificity”
24. Autism spectrum disorder OR "autism" AND "MRNA" AND (“classifier” OR “classification”)
25. Autism spectrum disorder OR "autism" AND "gene expression" AND “sensitivity”
26. Autism spectrum disorder OR "autism" AND "gene expression" AND “specificity”
27. Autism spectrum disorder OR "autism" AND "gene expression" AND (“classifier” OR “classification”)
28. Autism spectrum disorder OR "autism" AND "PCR" AND “sensitivity”
29. Autism spectrum disorder OR "autism" AND "PCR" AND “specificity”
30. Autism spectrum disorder OR "autism" AND "PCR" AND (“classifier” OR “classification”)
31. Autism spectrum disorder OR "autism" AND "metabolic" AND “sensitivity”
32. Autism spectrum disorder OR "autism" AND "metabolic" AND “specificity”
33. Autism spectrum disorder OR "autism" AND "metabolic" AND (“classifier” OR “classification”)
34. Autism spectrum disorder OR "autism" AND "urinary" AND “sensitivity”
35. Autism spectrum disorder OR "autism" AND "urinary" AND “specificity”
36. Autism spectrum disorder OR "autism" AND "urinary" AND (“classifier” OR “classification”)
37. Autism spectrum disorder OR "autism" AND "EEG" AND “sensitivity”
38. Autism spectrum disorder OR "autism" AND "EEG" AND “specificity”
39. Autism spectrum disorder OR "autism" AND "EEG" AND (“classifier” OR “classification”)
